# Impact of diabetes on COVID-19 patient health outcomes in a vulnerable racial minority community

**DOI:** 10.1371/journal.pone.0286252

**Published:** 2023-07-21

**Authors:** Stefan Hamaway, Uchechukwu Nwokoma, Michael Goldberg, Moro O. Salifu, Subhash Saha, Roosevelt Boursiquot

**Affiliations:** 1 Department of Medicine, State University of New York (SUNY) Downstate Health Sciences University, Brooklyn, NY, United States of America; 2 SUNY Downstate College of Medicine, Brooklyn, NY, United States of America; 3 Office of Diversity Education and Research, SUNY Downstate College of Medicine, Brooklyn, NY, United States of America; 4 Department of Neurology, State University of New York (SUNY) Downstate Health Sciences University, Brooklyn, NY, United States of America; 5 Division of Hospital Medicine, Department of Medicine, Icahn School of Medicine at Mount Sinai, New York, NY, United States of America; 6 Department of Internal Medicine, Northwell Staten Island University Hospital, Staten Island, NY, United States of America; Nazarbayev University School of Medicine, KAZAKHSTAN

## Abstract

**Background:**

Diabetes is a growing health concern in the United States and especially New York City. New York City subsequently became an epicenter for the coronavirus pandemic in the Spring of 2020. Previous studies suggest that diabetes is a risk factor for adverse outcomes in COVID-19.

**Objective:**

To investigate the association between diabetes and COVID-19 outcomes as well as assess other covariates that may impact health outcomes.

**Design:**

Retrospective cohort study of COVID-19 hospitalized patients from March to May, 2020.

**Participants:**

In total, 1805 patients were tested for COVID-19 and 778 tested positive for COVID-19. Patients were categorized into 2 groups: diabetes (measured by an Hba1c >6.5 or had a history of diabetes) and those without diabetes.

**Results:**

After controlling for other comorbidities, diabetes was associated with increased risk of mortality (aRR = 1.28, 95% CI 1.03–1.57, p = 0.0231) and discharge to tertiary care centers (aRR = 1.69, 95% CI 1.04–2.77, p = 0.036). compared to non-diabetes. Age and coronary artery disease (CAD) increased the risk of mortality among diabetic patients compared to patients with diabetes alone without CAD or advanced age. The diabetes cohort had more patients with resolving acute respiratory failure (62.2%), acute kidney injury secondary to COVID-19 (49.0%) and sepsis secondary to COVID-19 (30.1%).

**Conclusion:**

This investigation found that COVID-19 patients with diabetes had increased mortality, multiple complications at discharge, and increased rates of admission to a tertiary care center than those without diabetes suggesting a more severe and complicated disease course that required additional services at time of discharge.

## Introduction

A novel coronavirus disease 2019 (COVID-19), caused by SARS-CoV-2, began in Wuhan, China in December 2019 and has quickly spread throughout the world. The first confirmed case in the United States was reported on January 20, 2020, and the World Health Organization classified COVID-19 as a pandemic in March, 2020 [1]. Since the outbreak, the United States, and New York City in particular, emerged as an epicenter for severe acute respiratory syndrome coronavirus 2 (SARS-CoV-2) infections [2]. Black and Hispanic communities have been disproportionately more affected than their white counterparts. White communities received more testing while mortality in African Americans is higher [3]. Part of the difference can be explained by the risks taken by members in the respective communities as residents in African American communities are more likely to work as essential workers and did not have the luxury to stay at home and were thus put at an increased risk of contracting the disease [3]. Because of these health disparities, there is a particular interest in investigating COVID-19 outcomes in patients from minority inner-city backgrounds.

Several co-morbidities have been found to have an association with increased mortality from COVID-19 including cardiovascular disease, hypertension, diabetes, respiratory disease, and cancers [4]. Diabetes is highly prevalent in the U.S. and exerts a significant toll on the U.S. healthcare system. Approximately one in every 10 Americans (34.2 million) has diabetes and one in every five do not know that they have the disease [5]. The prevalence is higher in certain populations, including non-Hispanic blacks and Hispanics, who have also been reported to be disproportionately affected by COVID-19 [5, 6].

In particular, a 2021 meta-analysis by Saha et al. estimated that the mortality rate of hospitalized COVID-19 patients was 20% for those with comorbid diabetes, compared to 11% for those without [7]. The linkages between diabetes and COVID-19 disease burden were corroborated by Ando et al., who found evidence supporting that patients with comorbid diabetes or obesity were more likely to be hospitalized and suffer increased severity of disease [8]. They additionally found that white diabetic patients showed decreased mortality rates with usage of metformin and SGLT-2 inhibitors, but black and Asian patients did not show a mortality risk reduction, potentially due to well documented under prescription of these drugs towards these demographics [8, 9].

Overall, these studies show patients with diabetes were more susceptible to receiving mechanical ventilation, had increased admissions to the ICU, and had higher mortality compared to patients without diabetes [7, 10]. Several mechanisms have been proposed including chronic inflammation, increased coagulation activity, impaired immune responses, and potential direct pancreatic damage by SARS-CoV-2 [11]. However, there is limited information describing the association between diabetes and COVID-19 outcomes in vulnerable racial minority populations. The purpose of this study is to investigate the association between diabetes and COVID-19 outcomes in a community in Brooklyn, New York whose population is estimated to be 88% black and 15% diabetic and to investigate modulators that may worsen health outcomes among these diabetic patients [12].

## Methods

### Study design

This is a retrospective cohort study of patients tested for COVID-19 at SUNY Downstate Health Sciences University in New York, designated by Governor Andrew Cuomo as a COVID-only hospital. Testing was conducted via nasopharyngeal swab and subsequent reverse-transcription polymerase chain reaction (RT-PCR). Data were collected and deidentified for all hospitalized patients through the Healthbridge/Allscript Electronic Medical Health Record system. The Downstate institutional review board approved this study for publication.

### Consent

Institutional review board approval (IRB) was obtained (IRB# 1903048–1). The data was analyzed anonymously so a waiver for informed consent was obtained.

### Subjects

**Fig 1:**
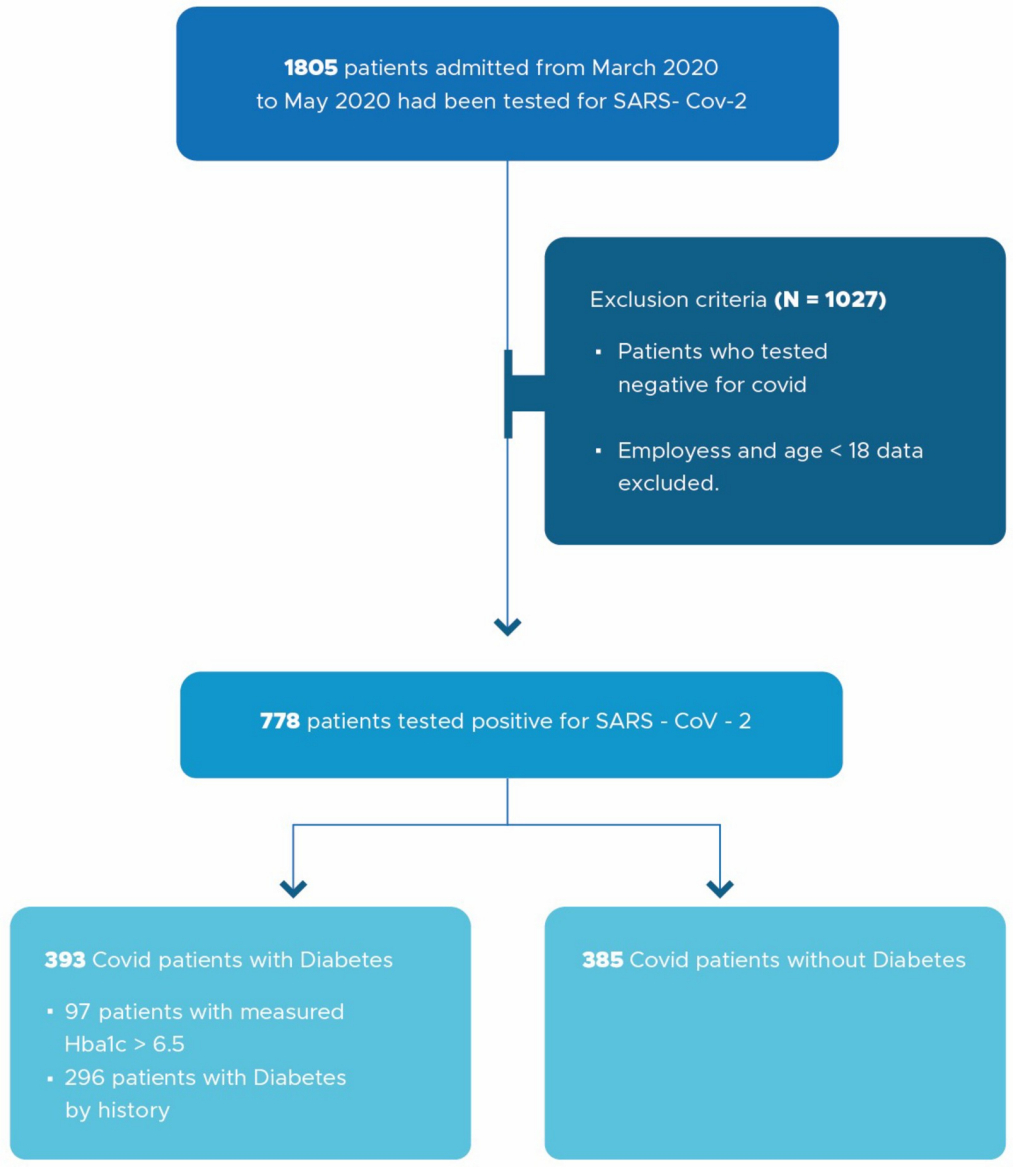
Workflow diagram of study participant selection criteria.

This study cohort had 1805 patients who were tested for COVID-19 and 778 patients were ultimately used for analysis. The population included patients 18 years of age or older who were admitted from March 2020 to May 2020. Patients were excluded from the study if their COVID-19 test was negative (Fig 1).

### Primary predictors

Diabetes was defined by patients who had been diagnosed with diabetes, using HbA1C over 6.5 or by history if HbA1C values were unavailable during this hospital admission. Patients were categorized into two groups: diabetics and non-diabetics.

### Covariates

Covariates were age, sex, coronary heart disease, congestive heart failure, chronic kidney disease, end-stage renal disease, hypertension, obesity, and hyperlipidemia.

### Outcomes

The primary outcome was in-hospital mortality, while the secondary outcome was discharge to a tertiary center for continued care. Tertiary care centers included skilled nursing facilities, inpatient rehabilitation service centers, and other acute care facilities where patients were admitted to receive additional services including nursing and rehabilitation therapy needs. Modulators that affected both in-hospital mortality and discharge to a tertiary care center among diabetic patients were then assessed and tabulated.

### Statistical analysis

All patients admitted during this timeframe that met the inclusion criteria were included in the study and separated by diabetes status. The association between diabetes and mortality and tertiary care center discharge was first analyzed among patients testing positive with COVID-19. Differences in demographics and clinical characteristics were compared between the diabetes and non-diabetes groups using chi-square for categorical variables and Kruskal-Wallis for continuous variables. The diabetic population was then restricted by age and other covariates to assess their association with mortality and tertiary care center discharge using log-binomial regression models. The covariates included in the models for mortality and discharge to a tertiary care center included: CAD, CHF, CKD, ESRD, hypertension, obesity, and hyperlipidemia. Asthma, cancer, and COPD were found to not be significant and were not incorporated into the models. Characteristics statistically significant at the 0.05 alpha level in bivariate analysis were included into the multivariable regression model. All statistical analysis was performed using SAS 9.4 software for Windows.

## Results

A total of 737 patients were included in the study with 381 patients with diabetes and 356 without (Table 1).

**Table 1:** Comparison of demographics of patients with diabetes and non-diabetes who tested positive for SARS-CoV-2.

Characteristic	Total n = 737	Diabetes n = 381 (51.70%)	Non-Diabetes n = 356 (48.30%)	p Value
**Age—mean ± SD**	65 ± 17	68 ± 12	62 ± 20	<0.0001
< 45	69 (9.47%)	11 (2.93%)	58 (16.43%)	
45–64	233 (31.69%)	121 (32.18%)	112 (31.73%)	
>65	427 (58.57%)	253 (64.89%)	183 (51.84%)	
**Male**	367 (49.80%)	191 (50.13%)	176 (49.44%)	0.8508
**Race**				0.5071
Black	654 (88.74%)	342 (89.76%)	312 (87.64%)	
W	45 (6.11%)	23 (6.04%)	22 (6.18%)	
Asian	4 (0.54%)	1 (0.26%)	3 (0.84%)	
American Indian—Alaska Native	1 (0.14%)	1 (0.26%)	0	
Undisclosed	33 (4.48%)	14 (3.67%)	19 (5.34%)	
**Comorbidities**				
Asthma	42 (5.70%)	24 (6.30%)	18 (5.06%)	0.467
Cancer	34 (4.61%)	15 (3.94%)	19 (5.34%)	0.3652
CAD	95 (12.89%)	63 (16.54%)	32 (8.99%)	0.0022
CHF	56 (7.60%)	39 (10.24%)	17 (4.78%)	0.0052
COPD	25 (3.39%)	15 (3.94%)	10 (2.81%)	0.3979
CKD	99 (13.43%)	66 (17.32%)	33 (9.27%)	0.0014
ESRD	87 (11.80%)	57 (14.96%)	30 (8.43%)	0.006
HTN	498 (67.57%)	301 (79.00%)	197 (55.34%)	<0.0001
Obesity (BMI>30)	107 (14.52%)	75 (19.69%)	32 (8.99%)	<0.0001
Hyperlipidemia	194 (26.32%)	129 (33.86%)	65 (18.26%)	<0.0001
**Outcomes**				<0.0001
Discharged Home	415 (56.31%)	181 (47.51%)	234 (65.73%)	
Transferred to Tertiary Care center	78 (10.58%)	53 (13.91%)	25 (7.02%)	
Mortality rate	244 (33.11%)	147 (38.58%)	97 (27.25%)	

CAD = Coronary Artery Disease; CHF = Congestive Heart Failure; COPD  =  Chronic Obstructive Pulmonary Disease; CKD = Chronic Kidney Disease; ESRD = End Stage Renal disease; HTN = Hypertension; BMI = Body Mass Index.

### Demographics

Of the 737 patients studied, 381 (51.7%) had a previous diagnosis of diabetes or a measured HbA1C over 6.5, while 356 did not have diabetes. The mean age of the cohort was 65±17 years. 367 (49.8%) were male, with 654 identifying as black (88.7%). Across the cohort, the most prevalent comorbidities included hypertension (n = 498, 67.6%), obesity (n = 107, 14.5%) chronic kidney disease (n = 99, 13.4%), coronary artery disease (n = 95, 12.9%), end stage renal disease (n = 87, 11.8%), and congestive heart failure (n = 56, 7.6%). Patients in the diabetes cohort were found to be significantly more likely to have any of the above comorbidities than the diabetes-free cohort (p<0.05) (Table 1).

### Mortality of diabetic patients with COVID-19 infections

Overall, diabetes status was found independently to be associated with an increased risk of mortality compared to non-diabetic patients (aRR = 1.28, 95% CI 1.03–1.57, p = 0.0231) (Table 2).

**Table 2:** Association of diabetes on in-hospital mortality of COVID-19 infected patients.

	Unadjusted or Crude Relative Risk	p-value	Adjusted Relative Risk	p-value
Diabetes	1.41 (1.15, 1.75)	0.0013	1.28 (1.03, 1.57)	0.0231

### Association of covariates that increased mortality among diabetic patients

Co-morbidities were analyzed to determine their impact on the relative risk for mortality in diabetic patients. When controlling for all other comorbidities, advanced age augmented the risk of mortality among diabetic patients compared to those diabetic patients less than 45 years old, with the 45–64 age group (aRR = 4.9, 95% CI 1.6–15.27, p = 0.0058) and 65+ age group (aRR = 9.44, 95% CI 3.1–28.8, p<0.0001) both associated with increased rates of mortality. Gender was also significant, where diabetic males were found to be at greater risk for mortality compared to diabetic females (aRR = 1.22, 95% CI 1.01–1.49, p = 0.039). Lastly, diabetic patients with CAD were associated with increased mortality compared to those with diabetes alone (aRR = 1.53, 95% CI 1.06–1.64, p = 0.0121) (Table 3).

**Table 3:** Association of covariates on in-hospital mortality of COVID-19 infected patients with diabetes.

Covariates	Unadjusted or Crude Relative Risk	p-value	Adjusted Relative Risk	p-value
Age:				
45–64	5.13 (1.65, 15.93)	0.0046	4.91 (1.58, 15.27)	0.0058
≥ 65	10.13 (3.33, 30.78)	<0.0001	9.44 (3.09, 28.80)	<0.0001
Male	1.33 (1.08, 1.64)	0.0066	1.22 (1.01, 1.49)	0.039
CAD	1.53 (1.20, 1.95)	0.0006	1.32 (1.06, 1.64)	0.0121
CHF	0.41 (0.22, 0.79)	0.0075	0.35 (0.19, 0.67)	0.0015
CKD	1.34 (1.04, 1.73)	0.0252	1.04 (0.82, 1.32)	0.7358
ESRD	0.85 (0.60, 1.21)	0.3702	0.98 (0.70, 1.36)	0.8958
HTN	0.85 (0.69, 1.05)	0.1332	Blank	Blank
Obesity (BMI>30)	0.79 (0.57, 1.10)	0.17	0.78 (0.56, 1.07)	0.1178
Hyperlipidemia	0.97 (0.77, 1.23)	0.8278	0.79 (0.64, 0.99)	0.0372

CAD = Coronary Artery Disease; CHF: Congestive Heart Failure; CKD = Chronic Kidney Disease; ESRD = End Stage Renal disease; HTN = Hypertension; BMI = Body Mass Index.

### Discharge to a tertiary care facility of diabetic patients with COVID-19 infections

Among COVID-19 survivors, the relative risk of diabetic patients transfer to a tertiary care facility on discharge was assessed (Table 4). Overall, diabetes status was found independently to be associated with an increased risk of discharge to tertiary care compared to patients without diabetes (aRR = 1.69, 95% CI 1.04–2.77, p = 0.036).

**Table 4:** Association of diabetes on discharge to a tertiary care center among patients surviving COVID-19 infection.

	Unadjusted or Crude Relative Risk	p-value	Adjusted Relative Risk	p-value
Diabetes	2.34 (1.51, 3.65)	0.0002	1.69 (1.04, 2.77)	0.0355

### Association of covariates that increased discharge to a tertiary care facility among diabetic patients

The relative risk for comorbidities in diabetic patients were analyzed to determine their association with transfer to a tertiary care facility among patients surviving COVID-19 infections (Table 5). Within this group, diabetic patients in the 65+ age group (aRR = 5.75, 95% CI 1.35–24.33, p = 0.017) and diabetic patients with CAD (aRR = 1.87, 95% CI 1.17–2.99, p = 0.0085) were both associated with an increased risk of discharge to a tertiary care facility compared to diabetic patients alone.

**Table 5:** Association of covariates on discharge to a tertiary care center among patients surviving COVID-19 infections.

Covariates	Unadjusted or Crude Relative Risk	p-value	Adjusted Relative Risk	p-value
Age:				
45–64	3.46 (0.83, 14.47)	0.0885	2.76 (0.67, 11.95)	0.1736
≥ 65	7.87 (1.97, 31.39)	0.0035	5.75 (1.35, 24.33)	0.0174
Male	0.85 (0.56, 1.29)	0.4481	0.83 (0.56, 1.25)	0.375
CAD	2.47 (1.57, 3.88)	<0.0001	1.87 (1.17, 2.99)	0.0085
CHF	1.36 (0.75, 2.47)	0.3059	0.93 (0.52, 1.66)	0.8102
CKD	1.12 (0.62, 2.06)	0.7024	0.86 (0.46, 1.59)	0.627
ESRD	1.66 (1.01, 2.72)	0.0465	1.29 (0.79, 2.09)	0.3036
HTN	2.43 (1.35, 4.36)	0.0029	1.09 (0.57, 2.09)	0.7977
Obesity (BMI>30)	1.60 (0.9997)	0.0502	1.28 (0.81, 2.00)	0.2924
Hyperlipidemia	1.46 (0.96, 2.23)	0.077	0.86 (0.56, 1.32)	0.4924

CAD = Coronary Artery Disease; CHF: Congestive Heart Failure; CKD = Chronic Kidney Disease; ESRD = End Stage Renal disease; HTN = Hypertension; BMI = Body Mass Index.

### Most common discharge diagnoses

**Fig 2:**
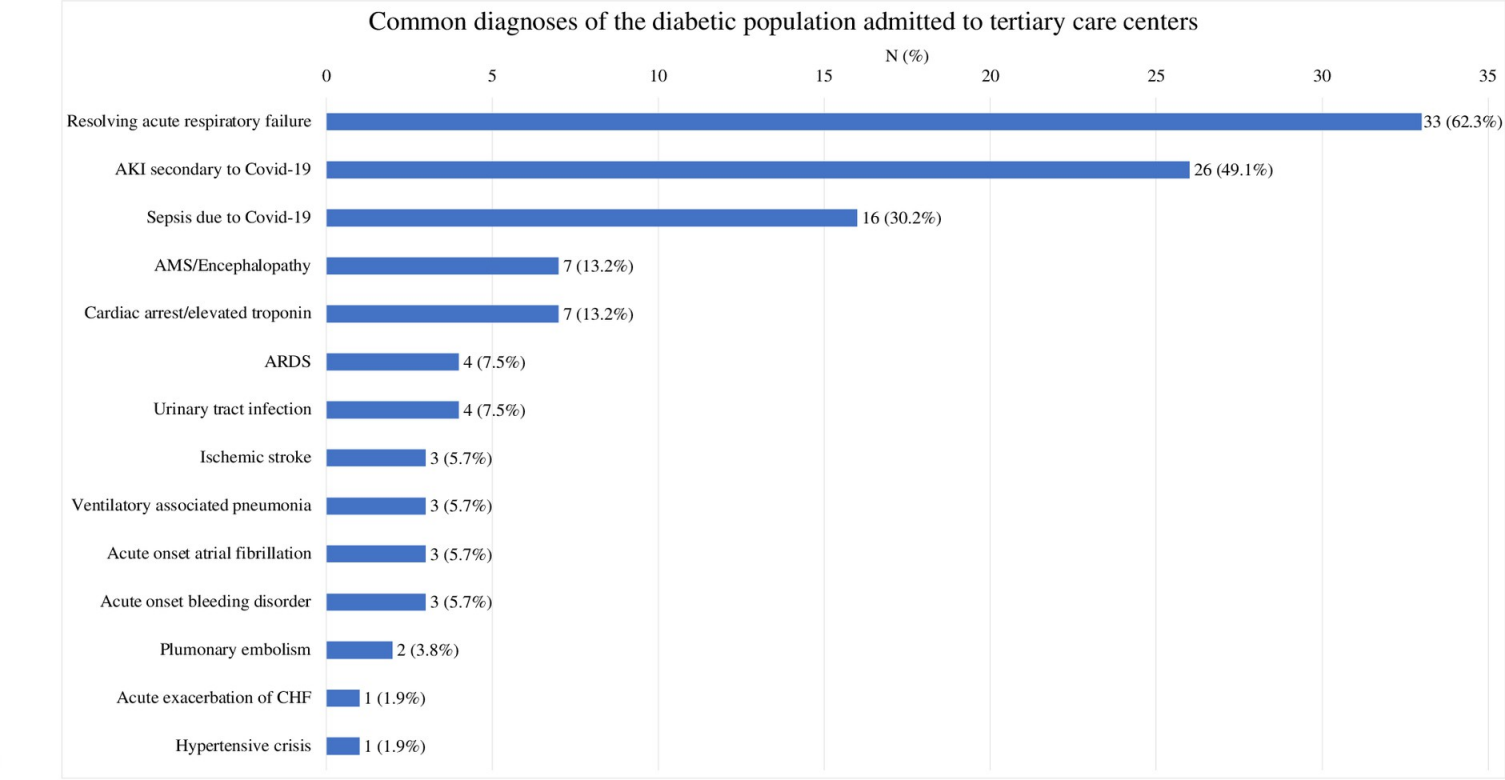
Most frequent discharge diagnoses of surviving diabetic patients on admission to tertiary care centers. AKI = Acute Kidney Injury; AMS: Altered Mental Status; ARDS = Acute Respiratory Distress Syndrome; CHF: Congestive Heart Failure.

Among the survivors, more diabetic patients were admitted to a tertiary care center (n = 53, 22.6%) compared to those without diabetes (n = 25, 9.7%).The most common discharge diagnoses of diabetic survivors being discharged to tertiary care facilities were then tabulated (Fig 2). Notably, the diabetes cohort had 33 (62.2%) patients with resolving acute respiratory failure, 26 (49.0%) with AKI secondary to COVID-19, and 16 (30.1%) with sepsis secondary to COVID-19, amongst other discharge diagnoses.

## Discussion

After adjusting for age, sex, coronary heart disease, congestive heart failure, chronic kidney disease, end-stage renal disease, hypertension, obesity, and hyperlipidemia, this study demonstrated that diabetes status is associated with a higher mortality rate in those who test positive for COVID-19, and that patients with diabetes were more likely to be discharged to a tertiary care center compared to those without diabetes. Several covariates were identified that potentiated this effect among diabetic patients including advanced age greater than 45 years old, CAD, and male sex that all increased their risk of mortality. Lastly, among survivors of COVID-19 with diabetes, patients frequently had resolving acute respiratory failure, AKI, or sepsis that required discharge to the tertiary care center.

The results of this study are consistent with previously published findings [13, 14]. This study found that diabetic patients had higher incidence of other comorbidities compared to non-diabetic patients, and that after controlling for all other comorbidities, age was found to significantly increase the risk of mortality among diabetic patients, with the 45–64 age group and 65+ age group both showing increased rates of mortality. Males were also found to be at greater risk for mortality among diabetic patients. Older diabetic male patients had the greatest overall mortality in the predominantly Black patient population. One study by Corona *et al* found that diabetes was the single most important cause of mortality after adjusting for confounders [15]. A study by Xie *et al* in New Orleans found that among black patients, metabolic syndrome using the clustering of hypertension, obesity, and diabetes was associated with significantly increased mortality and that metabolic syndrome as a cluster increased the odds of mortality compared to these comorbidities individually [16]. Our results are consistent with these findings with the increased risk of mortality among our predominantly black patient population with diabetes, but also expanded to assess other covariables that increased mortality including age and CAD. Our results contrast a study by Woolcott and Castilla-Bancayán in Mexico which found that the adjusted hazard ratio for death decreased with age (3.12 (95% CI 2.86–3.40) for patients 20–39 years of age vs 1.11 (95% CI 1.06–1.16) for patients 80 years of age or older) [13]. However, there were limitations to that study as it likely experienced confounding from unreported comorbidities in their inpatient population. Institutionalized adults over 80 years old are more likely to have neurodegenerative diseases which may not have been reported and was subsequently not controlled between study populations. Our study controlled for age with more frequent divisions so the association between diabetes and increased mortality was still seen in the younger 45–64 year old subgroup which is not as prone to having neurodegenerative disease confounder. A study by Shi *et al*. found that hypertension was independent association with increased risk of mortality in patients with diabetes [14]. Our study found that rather than hypertension, CAD was associated with increased risk of mortality in diabetic patients. This can be expected with diabetic patients because of the accelerated atherosclerotic process seen in diabetes with the chronic low-grade inflammation [17].

It is important to note the epidemiological factors that will impact these racial minority communities. Per the CDC, non-hispanic African Americans are about 56% more likely to be diagnosed with diabetes than non-Hispanic white Americans [5]. A study by Gupta *et al* that found that among black patients with COVID-19, 98% of hospitalized patients had one or more co-morbidities, with 56% having diabetes, and that these hospitalized patients faced a 48% mortality rate [18]. Their findings focused on CKD patients suggest that earlier escalation of care based on comorbidities and laboratory tests are crucial to improve outcomes in African-American patients. These racial minority communities have a history of distrust towards the health care system which may delay diagnosis by avoiding routine checkups which ultimately delays treatment [19, 20]. Many of the comorbidities included in our analysis are both easily identifiable and treatable, so there is a potential for improved outcomes of COVID-19 patients with diabetes with better screening and treatment of these marginalized patients.

Among COVID-19 survivors, diabetic patients compared to non-diabetic patients were associated with significantly high rates of requiring tertiary care facilities on discharge. Age and CAD modulated this effect, with diabetic patients in the 65+ age group and diabetic patients with comorbid CAD both associated with an increased risk of requiring discharge to a tertiary care facility including nursing homes and sub-acute rehabilitation facilities compared to patients with diabetes alone. While specific studies looking at discharge setting to indicate continued higher demands of care could not be found, other studies have looked at hospital readmissions. A study by Nematshahi *et al* found that diabetes was one of the most important predictors of hospital readmission (OR = 3.43, 95% CI 1.13–8.37) [21]. Another study by Atalla *et al* found that 57.9% of patients with diabetes were readmitted to the hospital compared to 33.3% without diabetes (p = .021) [22]. Those studies did not look at tertiary care facility usage on discharge but rather at readmissions. Our study assessed a patient’s immediate health status at discharge since it showed that patients required tertiary care facilities at discharge compared to their home environment at baseline (Fig 2). Thus, they had a more severe course of COVID-19 infection and at discharge required higher levels of care with a potentially longer recovery time post discharge in the facility before they could be rehabilitated and sent to their homes.

The diabetic cohort survivors had frequent discharges listed with acute respiratory failure (62.2%), AKI secondary to COVID-19 (49.0%), and sepsis secondary to COVID-19 (30.1%) (Fig 2). These patients had a more severe hospital since it was more likely to be complicated by acute respiratory failure, acute kidney injury and sepsis secondary to COVID-19. A study by Denson *et al*. reported similar findings that metabolic syndrome (obesity, prediabetes or diabetes, hypertension, and dyslipidemia) was associated with increased risk of ICU admission (adjusted odds ratio [aOR], 1.32 [95% CI, 1.14–1.53]), acute respiratory distress syndrome (aOR, 1.36 [95% CI, 1.12–1.66]), and mortality (aOR, 1.19 [95% CI, 1.08–1.31]) [23]. Another study by Khalili *et al*. found AKI develops in a large percentage of patients with COVID-19 and that patient with diabetes were especially more likely to have AKI compared to non-diabetic patients [24]. The diabetic patients had serious hospital courses because of their underlying comorbidity with various causes including a proinflammatory state in diabetes as well as immune dysfunction increasing their susceptibility to infection (Fig 2).

Experimental evidence found that patients with diabetes have chronic low-grade inflammation marked by higher levels of pro-inflammatory mediators including C-reactive protein, interleukin 6, and tumor necrosis factor alpha, as well as abnormal cytokine-secreting cells [17]. The chronic low-grade inflammation as well as the increased formation of advanced glycation end products can increase a patient’s susceptibility to infections [17, 25, 26]. There is also increased oxidative stress in diabetes that exacerbates the chronic inflammation seen in diabetes [26]. In addition to the intrinsic effects of diabetes on the cell and the molecular mechanism, diabetes is also linked with numerous comorbidities such as hypertension, CKD, and obesity [27]. The associated comorbidities as well as the diabetes related complications could contribute to the more severe course of COVID-19 observed. In addition, the increased inflammation seen with COVID-19, as well as treatment of COVID-19 with steroids can worsen a patient’s hyperglycemia thereby requiring increased insulin dosages.

There are limitations for this retrospective cohort study. Because of the retrospective nature of the study, data was limited to information already collected in the patient charts which were not designed explicitly for this study. The study was also conducted in the middle of the first wave of hospitalizations, so the analysis does not include subsequent infections or SARS-CoV-2 variants that have since appeared. As this study defined patients with diabetes with an HbA1C greater than 6.5 or with diabetes by history, the severity and control of their diabetes could not be assessed, which could potentially impact the dependent variables of the study. This can serve as the basis for future prospective investigations to investigate HbA1C levels on outcomes to assess if better diabetes control can normalize outcomes. Lastly, because the New York City area has numerous health care systems and patients seek emergent medical care close to their homes, it was not possible to assess readmission rates after patients were discharged to either their home or tertiary care centers as patients may not return to the same hospital system for follow up. It is possible that once discharged to a tertiary care center, patients with diabetes continue to experience worse health outcomes, but this could not be directly assessed. Despite these limitations, there is value to the study as it used an inner-city population to determine the association between diabetes and COVID-19 outcomes, which can be used by physicians to guide patient care and promote further investigation into this topic including prospective studies assessing improved healthcare screenings and earlier treatment initiation of these co-morbidities on outcomes in African-American communities.

## Conclusion

This investigation found that in our predominately black patient population 1. Patients with diabetes have higher incidences of comorbidities including CAD and that CAD potentiated the mortality among diabetic patients, 2. That patients with diabetes had more severe COVID infections (indicated by diagnoses of acute respiratory failure, AKI, and sepsis), and 3. That these patients had higher rates of discharge to a tertiary care center. Future studies should aim at assessing the severity and duration of diabetes and glycemic control on COVID-19 outcomes as better control of a patient’s underlying diabetes could potentially improve outcomes in African-American patients. The study is useful as it can be used by healthcare professions to educate patients with diabetes about the potential complications following COVID-19 infection.

## Supporting information

S1 Dataset (XLSX)
